# Antiviral Activity of 3D, a Butene Lactone Derivative Against Influenza A Virus In Vitro and In Vivo

**DOI:** 10.3390/v13020278

**Published:** 2021-02-11

**Authors:** Zhenya Wang, Jieyu Fang, Jiao Luo, Duoduo Hou, Yayun Tan, Zichen Gu, Yongzhuang Ge, Lu Mao, Luyang Liu, Hongmin Liu, Zhanyong Wei, Haiwei Xu

**Affiliations:** 1 Collaborative Innovation Center of New Drug Research and Safety Evaluation, Key Laboratory of Advanced Drug Preparation Technologies, Ministry of Education, Zhengzhou University, Zhengzhou 450001, China; zhenyawang@zzu.edu.cn (Z.W.); fangjieyu1996@163.com (J.F.); luojiao8891@163.com (J.L.); hdd950712@163.com (D.H.); tanyayun789@163.com (Y.T.); guzichen22@163.com (Z.G.); yongzhuangge@163.com (Y.G.); maolu2020@163.com (L.M.); Liuluyang2021@163.com (L.L.); liuhm@zzu.edu.cn (H.L.); 2 Key Laboratory of “Runliang” Antiviral Medicines Research and Development, Institute of Drug Discovery & Development, Zhengzhou University, Zhengzhou 450001, China; 3 College of Animal Science and Veterinary Medicine, Henan Agricultural University, Zhengzhou 450002, China; weizhanyong@henau.edu.cn

**Keywords:** butenolide, antiviral activity, influenza A virus, apoptosis, cytokines

## Abstract

Influenza A virus is a highly variable and contagious respiratory pathogen that can cause annual epidemics and it poses an enormous threat to public health. Therefore, there is an urgent need for a new generation of antiviral drugs to combat the emergence of drug-resistant strains of the influenza virus. A novel series of butene lactone derivatives were screened and the compound 3D was selected, as it exhibited in vitro potential antiviral activity against A/Weiss/43 H1N1 virus with low toxicity. In addition, 3D dose-dependently inhibited the viral replication, expression of viral mRNA and viral proteins. 3D exerted a suppressive effect on A/Virginia/ATCC2/2009 H1N1 and A/California/2/2014 H3N2 in vitro. The time-of-addition analysis indicated that 3D suppressed H1N1 in the early stage of its life cycle. A/Weiss/43 H1N1-induced apoptosis in A549 cells was reduced by 3D via the mitochondrial apoptosis pathway. 3D could decrease the production of H1N1-induced pro-inflammatory cytokines that are induced by H1N1 in vitro and in vivo. The administration of 3D reduced lung lesions and virus load in vivo. These results suggest that 3D, which is a butene lactone derivative, is a promising agent for the treatment of influenza A virus infection.

## 1. Introduction

Influenza is a serious global public health problem [1]. It is a contagious pandemic respiratory disease that causes severe morbidity and mortality worldwide [2]. The influenza virus (IAV) can cause frequent seasonal epidemics with severe outcomes. In the last two decades, the pandemic influenza A H1N1 2009 virus (A/2009/H1N1) caused the first pandemic influenza of the new millennium, which affected over 214 countries and it caused over 18,400 deaths. In 2013, infection with avian influenza A H7N9 virus caused severe pneumonia in patients and it did not respond to typical or atypical antimicrobial treatment [3,4]. Therefore, vaccinations and antiviral drugs are required for the prevention and treatment of influenza. However, antigenic drift and shift, safety, and the emergence of seasonal viruses make the use of vaccinations difficult [5]. M2 inhibitors (rimantadine and amantadine) have been historically approved for treatment and prevention of IAV infection. CDC has not recommended the use of rimantadine and amantadine for recently circulating influenza viruses because many strains of influenza virus, including the 2009 H1N1 influenza virus, are now resistant to these drugs [6]. There are two classes of drugs recommended by CDC for use against recently circulating influenza viruses, including neuraminidase inhibitors (peramivir, zanamivir, and oseltamivir phosphate) and PA inhibitor (baloxavir) [7,8]. However, several recent studies have reported efficacies of NA inhibitors were limited due to the emergence of drug-resistant IAV strains [9,10,11]. These problems pose challenges for the treatment or prevention of influenza. It is urgent to develop and identify novel drugs for controlling the drug-resistant strains and mitigation of pandemic situations.

Butenolides are found in a variety of natural product scaffolds, such as andrographolide [12], aspulvione [13], and uncinine [14]. In addition, butenolides present a series of biologically active properties, including anti-virus, anti-tumor, anti-bacterium, and anti-inflammation [15,16,17]. Andrographolide exhibits antiviral effects against H1N1 by targeting the retinoic acid-inducible gene-I (RIG-I) receptors signaling pathway [18]. We are interested in the research of butene lactone compounds against influenza virus, according to previous studies on the synthesis and biological activity of butenolides [19,20]. For this reason, we synthesized a series of novel butene lactone compounds based on the lead compound andrographolide and assayed their activities against IAV. Among these compounds, 3D exhibited the best antiviral activity against IAV and was selected for the current study. The antiviral mechanism of butene lactone derivatives, including 3D, has rarely been elucidated. In this study, we examined the effect of 3D and found its broad-spectrum activity against IAV in vivo and in vitro. We then investigated the mechanism of 3D action on RIG-I-mediated pro-inflammatory response and IAV-induced apoptosis in host cells.

## 2. Materials and Methods

### 2.1. Compounds

Butenolide derivatives were synthesized and provided by Dr. Haiwei Xu’s Research Group. Ribavirin (RBV), which is an antiviral agent against a broad spectrum of viruses, was purchased from Zahn Chemical Technology Co. (Shanghai, China). The NA inhibitor Oseltamivir acid (OC) and its prodrug Oseltamivir (OS) were obtained from APExBIO Technology LLC (Houston, TX, USA).

### 2.2. Cells and Virus

Madin–Darby canine kidney (MDCK) cells and human lung adenocarcinoma (A549) cells were purchased from Chinese Academy of Sciences Cell Bank (Shanghai, China). Cells were cultivated in Dulbecco’s modified Eagle’s medium (DMEM; Hyclone, Logan, USA) containing 10% fetal bovine serum (FBS; Gibco, Grand Island, USA) at 37 °C in a humidified atmosphere containing 5% CO_2_. The influenza strains A/Virginia/ATCC2/2009 H1N1 and A/California/2/2014 H3N2 were purchased from the American Tissue Culture Collection (ATCC; Manassas, VA, USA). The A/Weiss/43 H1N1 virus was obtained from the China Center for Type Culture Collection (Wuhan, China). All of the viruses were propagated in MDCK cells at 37 °C. The viruses were harvested from cell culture supernatant by centrifuging virus growth medium at 1500× *g* for 10 min. and stocked at −70 °C until use. The virus titer was determined by the median tissue culture infective dose (TCID_50_).

### 2.3. Mice

Special-pathogen-free female BALB/c mice that were six weeks old and weighed 18–22 g were purchased from Beijing Vital River Laboratory Animal Technology Co., Ltd. (Beijing, China). All of the mice were housed under specific-pathogen-free conditions in a 12 h light/dark cycle, where the temperature was controlled at 22 ± 2 °C. During the experiment, all of the protocols were complied with the requirements in the Guide for the Care and Use of Laboratory Animals and approved by the Experimental Animal Ethics Committee of Zhengzhou University.

### 2.4. Cytotoxicity Assay

The 3-[4, 5-dimethylthiazol-2-yl]-2, 5-diphenyltetrazolium bromide (MTT; Biotopped, Beijing, China) assay was performed in order to assess the cytotoxicity of compounds. A549 or MDCK cells were seeded in 96-well plates at 1 × 10^4^ cells per well. After 24 h, cell growth medium was removed and cells were washed three times with phosphate-buffered saline (PBS). Subsequently, cells were treated by cell maintenance medium containing a series of concentrations of compounds and no compound as control for 72 h at 37 °C in a humidified 5% CO_2_ atmosphere. Afterwards, 20 µL of MTT reagent (5 mg/mL) was added to each well and incubated at 37 °C for 4 h. The supernatants were gently aspirated and added 150 µL dimethyl sulfoxide (DMSO) to each well. The plates were shaken on a decolorizing shaker for 10 min. for dissolving the formazan product at room temperature. The absorbance (OD) at 490 nm were measured using a microplate reader. The cell viability rate was calculated, as follows:

Cell viability (%) = [the average OD value of the compound-treated cells/control average OD value] × 100%
(1)

The 50% cytotoxic concentration (CC_50_) was calculated using SPSS software. At least three independent experiments were required.

### 2.5. Antiviral Activity Assay

A549 and MDCK cells were seeded in 96-well plates at 1 × 10^4^ cells per well and then cultured at 37 °C for 18–24 h. When cells were 80–90% confluent, removed growth medium and washed three times by using PBS. The cells were inoculated with viruses at 100TCID50 per well in FBS-free DMEM containing 2 µg/mL TPCK-trypsin for 2 h at 37 °C. After absorption, medium was discarded and washed with PBS. Subsequently, DMEM containing various concentrations of compounds and 2 µg/mL TPCK-trypsin were added into each well. Meanwhile, the normal controls (with no viruses and compounds) and virus controls (without compounds) were set up.

After 72 h, MTT assays were used in order to measure the absorbance in MDCK cells. The inhibition rate (%) was calculated according to the following formula:

Inhibition (%) = [(the average OD value of drug-treated group − the average OD value of virus controls)/(the average OD value of normal controls − the average OD value of virus controls)] × 100%(2)

A549 cells and medium were collected, frozen, and thawed twice, and then centrifuged to obtain supernatants. Subsequently, the inhibition of compound in A549 cells was determined by the changes of viral titers that were measured by TCID_50_.

### 2.6. Inhibitory Effects of 3D on Different Stages of Viral Infection

We conducted this experiment to determine which stage the compounds worked. The MDCK cells were treated with various concentrations (3.125, 6.25, 12.5, 25, 50, and 100 µM) before viral infection for 2 h (protocol 1), during viral infection for 2 h (protocol 2) and after viral infection (protocol 3). After 72 h inoculation, MTT was added into the plates and the inhibition rates of compounds were assessed.

### 2.7. Quantitative Real-Time PCR Assay (qRT-PCR)

The levels of different genes were detected by qRT-PCR. The total RNA from cells or lung tissues was extracted according to the instructions of Ultrapure RNA Kit (CW, Beijing, China). 1 µg of RNA was reverse transcribed into cDNA by using Thermo Scientific Revert Aid First Strand cDNA Synthesis Kit (Thermo, Waltham, MA, USA). qRT-PCR was performed using QuantiNovaTM SYBR^®^ Green PCR Kit (Qiagen, Hilden, Germany). The primer sequences were designed and synthesized using the corresponding cDNA as the templates. Supplementary Table S1 lists the primer sequences (BGI, Shenzhen, China). Additionally, the qRT-PCR cycling conditions were: 95 °C (2 min.); 40 cycles of 95 °C (5 s), 60 °C (10 s), and melt curve (65 to 95 °C, increment 0.5 °C for 5 s). Finally, GAPDH was used as an internal reference gene and the relative expression of the gene was calculated by the cyclic threshold (2^−ΔΔCt^) method.

### 2.8. Time-of-Addition Assay

A time-of-addition assay was performed in order to elucidate the special time when viral replication was interfered by 3D. In brief, the MDCK cells were treated with 3D (50 µM) at indicated time periods ((−2)–0, 0–2, 2–4, 4–6, 6–10). Virus control was treated with maintained medium without 3D. Subsequently, at 10 h post infection, MDCK cells were collected and RNA was extracted. The relative expression of M gene was detected by qRT-PCR to elucidate the time 3D taken effect.

### 2.9. Apoptosis Assay

Apoptosis was detected by flow cytometer with Annexin V-FITC/PI staining. A549 cells were seeded in six-well plates at a density of 2 × 10^5^ cells per well. When cells grew about 80% monolayer, the cells were infected or mock-infected with IAV (100 TCID_50_) for 2 h. Subsequently, maintained medium with different concentrations of 3D or without compounds was added to the six-well plates. After compounds treatment for 24 h, all of the operations were performed according to the instructions of the Annexin V-FITC/PI Apoptosis Detection Kit (BestBio, Shanghai, China). Briefly, the cells were dissociated with trypsin without EDTA, suspended by 400 µL binding buffer, and stained with 5 µL Annexin V-FITC for 15 min. and 10 µL PI for 5 min. Subsequently, cell suspension was analyzed with flow cytometer immediately and the proportion of apoptotic cells for each sample was presented.

### 2.10. Western Blotting Assay

A549 cells that were infected with IAV were treated by different concentrations of 3D (30 µM, 50 µM, and 80 µM). After 24 h infection, the cells were collected, added into RIPA buffer, and lysed for 30 min. on ice After centrifugation at 12,000 rpm for 12 min, the supernatants were aspired, and protein concentrations were quantified by BCA kit. The protein was denatured at 100 °C for 10 min. Equal amounts of protein (30 µg) were loaded in every well, separated by 10% sodium dodecyl sulfate-polyacrylamide gel electrophoresis (SDS-PAGE), and then transferred to nitrocellulose filter membrane. The membranes were blocked with 5% skimmed milk for 2 h and incubated with primary antibodies overnight at 4 °C. Antibodies against cleaved caspase-3 (CST), cleaved caspase-7 (CST), cleaved caspase-9 (CST), cleaved PARP (CST), Bcl2 (CST), Bax (CST), GAPDH (ZSJQ-Bio), and viral M2 (Abcam) were diluted at a ratio of 1:1000. The membranes were washed with TBST three times to remove the unbound primary antibodies. Subsequently, horseradish peroxidase (HRP)-conjugated goat anti-mouse or anti-rabbit secondary antibodies (CST, 1:3000) were added on the membranes for 2 h at room temperature. Finally, membranes were covered by chemiluminescent (ECL) substrate and the protein bands were detected. The images were analyzed by ImageJ software.

### 2.11. In Vivo Experiments

Six-week female BALB/c mice were randomly divided into seven groups, as follows, the normal control group, the virus control group, the 3D high-dose group (100 mg/kg/d), the 3D middle-dose group (50 mg/kg/d), the 3D low-dose group (25 mg/kg/d), the oseltamivir group (50 mg/kg/d), and the ribavirin group (50 mg/kg/d). Each group had 18 mice. After the mice acclimatization for three days, mice, except normal control mice, were intranasally infected with 40 µL of A/Weiss/43 H1N1 virus while normal control mice were intranasally treated with 40 µL of normal saline. After 4 h post infection, the compounds-treated mice were intragastrically administered with different compounds once a day for six consecutive days. As for control mice, were given saline once daily for six days. The symptoms of the mice were observed, and the recorded daily and body weight was monitored. Mice were euthanized at two, four, and six days post-infection. Lungs were morphologically observed, weighted, and divided into two parts, one for histopathological, immunohistochemistry, and the other one for qRT-PCR. Serum was collected for subsequent experiments. The lung index and the inhibition radio were calculated by using the following formula:

Lung index = lung weight (g)/body weight (g) × 100%(3)

Inhibition = (the lung index of virus group − the lung index of drug-treated group)/the lung index of virus group × 100%(4)

### 2.12. Histopathology and Immunohistochemistry

Lung tissues from mice at six days post-infection were fixed with 4% polyformaldehyde, embedded in paraffin, and then sliced for histopathology and immunohistochemistry. The tissue sections were dewaxed, hydrated, stained with hematoxylin and eosin, and dehydrated. Finally, the lung tissue lesions were observed under the microscope. For immunohistochemistry, the sections were dewaxed, hydrated, repaired, blocked, treated with anti-influenza A virus nucleoprotein (NP) antibody and HRP-labelled antirabbit antibodies, stained with 3,3-diaminobenzidin (DAB) and hematoxylin, and dehydrated. Subsequently, the expression of viral antigen in the lung tissue was observed.

### 2.13. Statistical Analysis

All of the statistical analyses were performed using GraphPad Prism 8 and IBM SPSS Statistics 21. At least three independent experiments were required. The experimental results were presented as the mean ± standard deviation (SD). Statistical differences between the groups were assessed using the student’s *t*-test, followed by one-way ANOVA. The results were considered to be statistically significant at *p* < 0.05 (*/^#^), *p* < 0.01 (**/^##^).

## 3. Results

### 3.1. The Synthetic Route of Compounds and Their Antiviral Activity against A/Weiss/43 H1N1 In Vitro

**Figure 1:**
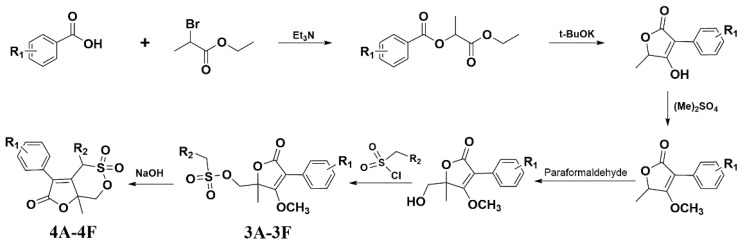
Synthesis route for compounds.

Figure 1 shows the synthetic route of the series of novel butene lactone compounds. All of the synthetic compounds were evaluated by antiviral activity against A/Weiss/43 H1N1 in MDCK cells (Table 1). Compounds (3D, 3E, 4A, 4C, and 4E) presented the antiviral activity against A/Weiss/43 H1N1. Among them, 3D was the best one, which had an EC_50_ of 12.30 µM with low toxicity.

### 3.2. Antiviral Activity of 3D against Influenza A Virus In Vitro

**Figure 2:**
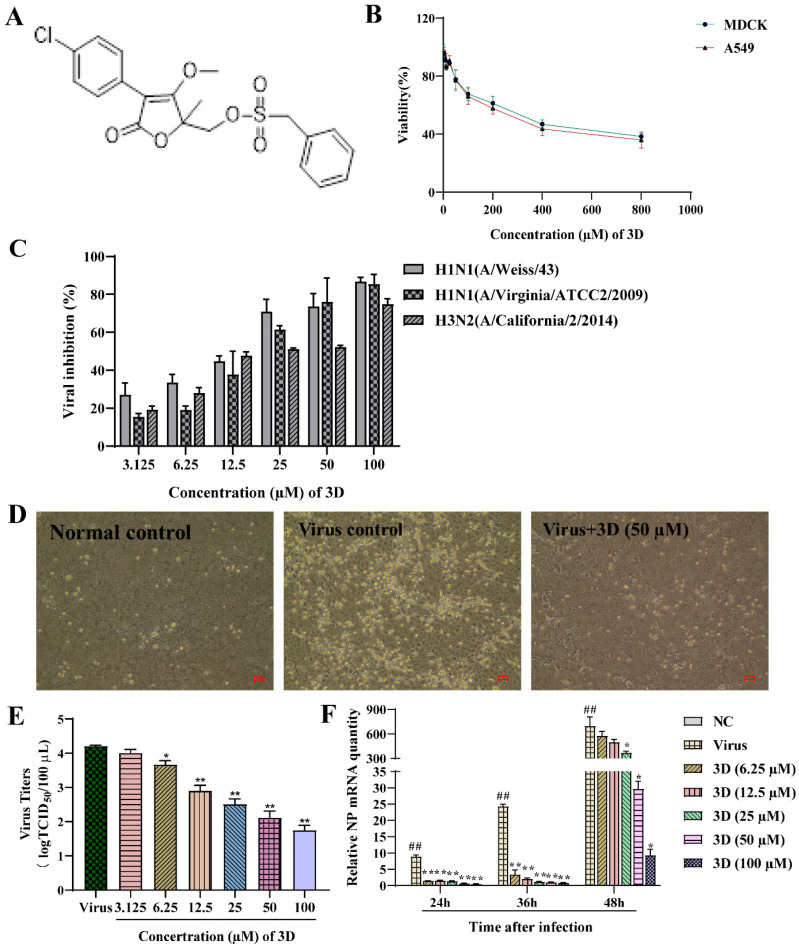
Inhibition of influenza virus infection by 3D, a butene lactone compound. (**A**) The chemical structure of 3D. (**B**) Cytotoxic effect of 3D on MDCK and A549 cells. Cells were treated with different concentrations of 3D for 72 h and cell viability was measured by 3-[4, 5-dimethylthiazol-2-yl]-2, 5-diphenyltetrazolium bromide (MTT) assay. (**C**) MDCK cells were infected with influenza A virus (A/Weiss/43 H1N1, A/Virginia/ATCC2/2009 H1N1 and A/California/2/2014 H3N2; 100 TCID_50_) and then treated with indicated concentrations of 3D for 72 h. Then, MTT assay was performed to determine the viability of these cells. (**D**) MDCK cells were mock-infected or infected with A/Weiss/43 H1N1. The image was acquired after incubation with 3D (50 µM) for 24 h (100×). (**E**) A549 cells infected with A/Weiss/43 H1N1 were treated with different concentrations of 3D for 72 h. Viral titers in the medium were analyzed by measuring the TCID50. (**F**) MDCK cells were infected with A/Weiss/43 H1N1 and treated with different concentrations of 3D. The expression of NP mRNA was detected by qRT-PCR at 24, 36, and 48 h post-infection (*x*-axis). The data are shown as the mean ± SD. (^##^ *p* < 0.01 as compared with the normal control; * *p* < 0.05, ** *p* < 0.01 compared with the virus control).

The non-toxic concentration range of 3D in MDCK and A549 cells was determined through MTT assay. 3D had small cytotoxic effects on A549 and MDCK cells, as the cell viability was approximately 40%, even at 800 µM (Figure 2B). The CC_50_ values of 3D on MDCK and A549 cells were calculated by SPSS software, which were 365.02 ± 1.58 µM and 308.76 ± 9.96 µM, respectively.

The antiviral activities of different concentration of 3D (3.125–100 µM) on different IAV strains, including A/Weiss/43 H1N1, A/Virginia/ATCC2/2009 H1N1 and A/California/2/2014 H3N2, were assayed on MDCK cells oseltamivir and ribavirin were used as positive controls. 3D could efficiently inhibit infection of IAV with EC_50_ values from 12.30 to 24.09 µM, as mentioned in Table 2. Additionally, 3D inhibited these three virus strains in a dose-dependent manner (Figure 2C). Microscopic observation revealed that 3D could effectively reduce the cytopathic effect of A/Weiss/43 H1N1 on MDCK cells (Figure 2D). Furthermore, virus titers of A549 cells significantly decreased after the treatment of 3D in a dose-dependent manner (Figure 2E). Therefore, the results indicate that 3D exerts a broad- spectrum anti-IAV activity without significant cytotoxicity.

The levels of IAV nucleoprotein (NP) mRNA were detected by qRT-PCR at 24, 36, and 48 h post-infection after treatment with different concentrations of 3D (6.25–100 µM). The results demonstrate a decrease in the levels of NP mRNA in IAV-infected MDCK cells (Figure 2F), indicating that 3D could effectively inhibit the viral replications cycle.

### 3.3. The Effects of 3D on Different Stages of Viral Infection

**Figure 3:**
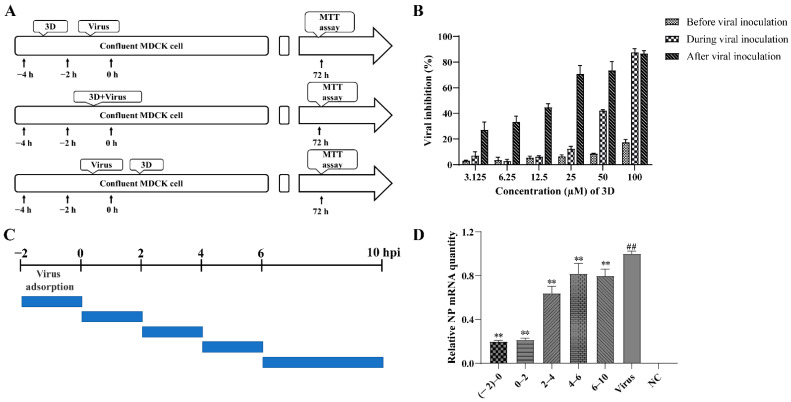
Inhibition of influenza A virus replication by 3D. (**A**,**B**) MDCK cells were treated with various concentrations of 3D before viral adsorption for 2 h, during viral adsorption for 2 h and after viral adsorption. The inhibition rates were determined by MTT assay at 72 h post-infection. (**C**,**D**) The infected MDCK cells were treated with 3D at indicated time intervals. At 10 h post-infection, cells were harvested and the expression level of viral NP mRNA was analyzed by qRT-PCR. The data are shown as the mean ± SD. (^##^ *p* < 0.01 compared with the normal control; ** *p* < 0.01 compared with the virus control).

In order to examine the stages at which 3D interferes with viral replication, 3D was added at varying concentrations into the medium before, during, or after viral adsorption, and the cell viability was measured by MTT assay at 72 h post-infection (Figure 3A). When added before viral adsorption, 3D had almost no inhibitory effects on viral replication and a slight inhibitory effect was observed when added during viral inoculation. When 3D was added after viral adsorption, a significant protective effect was observed on the infected MDCK cells (Figure 3B). These results show that the activity of 3D against IAV was mainly manifested as a therapeutic effect through different dosing methods.

A time-of-addition assay was performed to further understand the stage of the single-round replication cycle at which 3D works, as shown in Figure 3C. IAV-infected MDCK cells were added at different time points into DMEM medium containing 50 µM 3D, and the levels of NP mRNA were tested at 10 h post-infection. The results confirm that the levels of NP mRNA were significantly suppressed with 3D treatment, especially at (−2)–0, and 0–2 periods of the viral replication cycle (Figure 3D), indicating the inhibitory effect of 3D at the early stage of the viral cycle.

### 3.4. Effects of 3D on IAV-Induced Apoptosis and the Mitochondrial Apoptosis Pathway in A549 Cells

**Figure 4:**
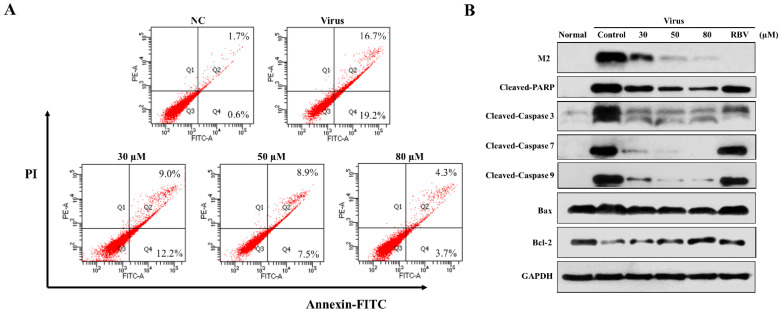
Effect of 3D on apoptosis induced by H1N1. A549 cells were mock-infected or infected with A/Weiss/43 H1N1 and treated with 3D (30, 50, and 80 µM) for 24 h. (**A**) Apoptosis was analyzed by flow cytometry using Annexin V-FITC/PI staining. (**B**) Cell lysates were collected and the indicated proteins were detected by western blot assay. For positive control, the indicated cells were treated with ribavirin (100 µM).

Pandemic influenza A (H1N1) virus can induce apoptosis in human airway epithelial cells due to caspase-3 activation [21,22]. In order to explore the effect of 3D on apoptosis, the infected A549 cells were treated with various concentrations of 3D, and apoptosis was detected by flow cytometry at 24 h post-infection. The apoptosis rate of virus control increased markedly, due to viral infection (32.85 ± 3.05%) as compared with normal control (2.30 ± 0.75%). The apoptosis rates were, respectively, 21.93 ± 0.97%, 15.17 ± 2.87%, and 7.23 ± 1.30% at 30 µM, 50 µM, 80 µM concentrations (Figure 4A). Thus, 3D evidently reduced the IAV-induced apoptosis in A549 cells.

We estimated the changes in the levels of apoptosis-related proteins in infected A549 cells with drug stimulation to further clarify the mechanism of apoptotic inhibition by 3D. Monolayer A549 cells were mock-infected or infected with A/Weiss/43 H1N1 virus and then treated with 3D or blank medium. The levels of cleaved caspase-3, cleaved caspase-7, cleaved caspase-9, and cleaved PARP visibly decreased after 3D treatment in a dose-dependent manner. Moreover, the expression of influenza M2 protein was inhibited by 3D, confirming a good anti-IAV effect of 3D through the inhibition of apoptosis proteins (Figure 4B).

The mitochondrial apoptotic pathway is activated during influenza virus infection. Mitochondria are believed to play a central role in the process of apoptosis. The expressions of Bax (a pro-apoptotic protein) and Bcl-2 (an anti-apoptotic protein) were tested to explore whether 3D affects apoptosis by mitochondrial pathway. We found that IAV-infection increased the ratio of Bax/Bcl-2, while 3D treatment reduced this upregulation of Bax and Bcl-2 in A549 cells (Figure 4B). These observations indicate that 3D reduced IAV-induced apoptosis through the mitochondrial apoptosis pathway in A549 cells.

### 3.5. Effects of 3D on the Production of Pro-Inflammatory Cytokines Induced by H1N1 Infection

**Figure 5:**
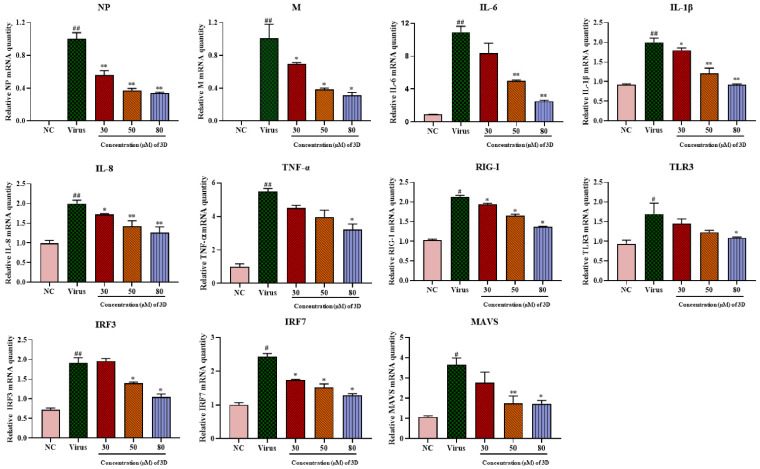
Effects of 3D on the expression of inflammatory mediators in A549 cells. Infected A549 cells were incubated in the absence or presence of the indicated concentrations of 3D (the *X*-axis) for 24 h. Subsequently, total RNA was extracted, and the qRT-PCR assay was performed to figure out the expression level of mRNA. (^#^ *p* < 0.05, ^##^ *p* < 0.01 as compared with the normal control; * *p* < 0.05, ** *p* < 0.01 compared with the virus control).

IAV infection can lead to mass production of pro-inflammatory factors, causing severe lung damage and aggravating the severity of the disease. A549 cells were infected with A/Weiss/43 H1N1 virus, and the expression levels of pro-inflammatory cytokines were determined by qRT-PCR at 24 h post-infection. IAV infection significantly up-regulated the mRNA levels of viral genes and pro-inflammatory cytokines, including interleukin (IL-1β), IL-6, IL-8, and tumor necrosis factor- α (TNF-α). As expected, 3D could decrease the levels of viral NP and M mRNA, and it significantly decreased the mRNA expression of these cytokines and chemokines in a dose-dependent manner (Figure 5).

The mRNA expression of RIG-I and its downstream signaling molecules, including mitochondrial antiviral signaling protein (MAVS), toll-like receptor 3 (TLR3), interferon regulatory factor 3 (IRF3), and IRF7, increased significantly in infected cells as compared with those in normal cells (Figure 5) and their levels decreased after 3D treatment. Taken together, these results suggest the inhibitory effect of 3D on the production of pro-inflammatory cytokines via the decrease in RIG-I expression and its downstream signaling pathway.

### 3.6. Therapeutic Efficiency of 3D against A/Weiss/43 H1N1 In Vivo

**Figure 6:**
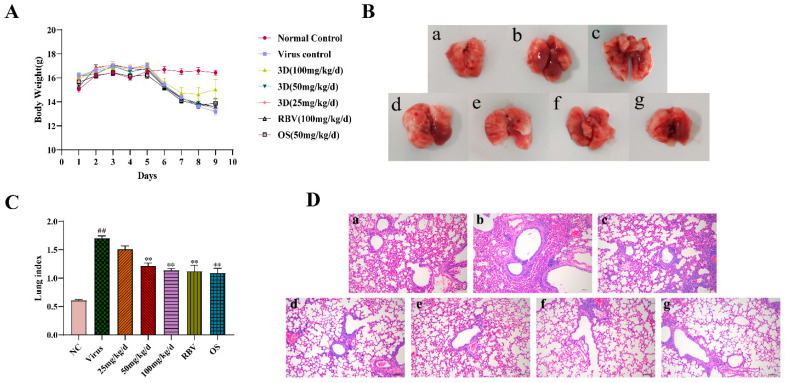
Evaluation of therapeutic efficacy of 3D in vivo. After three days for adaption, mice were mock-infected or infected with A/Weiss/43 H1N1. Mice were intragastrically administered with different compounds once a day for six consecutive days. (**A**) Body weight was monitored every day. (**B**) At six days post-infection, lungs were harvested, observed, and weighed. (**C**) The lung indexes were calculated and analyzed. (**D**) Photomicrographs of histopathology in lung tissue with HE staining were recorded (100×). Data are shown as the mean ± SD. (^##^ *p* < 0.01 compared with the normal control; ** *p* < 0.01 compared with the virus control.) (**B**) and (**D**) samples from (a) the normal control group; (b) the virus control group; (c) the 3D low-dose group, (d) the 3D middle-dose group, (e) the 3D high-dose group, (f) the ribavirin group, and (g) the oseltamivir group.

An infected mice model was used to further explore the therapeutic efficacy of 3D against IAV in vivo. After infection, the changes in body weight and clinical symptoms were observed every day. On the third day post-infection, mice of the virus group showed clinical symptoms, such as slow and heavy breathing, bradykinesia, poor appetite, weight loss, and shaggy fur. Treatments with 3D, oseltamivir, and ribavirin could relieve these symptoms (the change in body weight is shown in Figure 6A).

On the sixth day after infection, intact lung tissues were harvested, and the image was acquired. The lung tissues of the normal control group were pink, while a portion of the lung tissues of the virus control group appeared obviously dark red, indicating severe lung hyperemia and lesions. The range and degree of damaged lung tissues were reduced with the treatment of control drugs and 3D. In addition, the inhibition of dark red lung tissues by 3D occurred in a dose-dependent manner (Figure 6B). Accordingly, the lung indexes of mice in the virus control group were significantly increased when compared with that of the normal control group. The lung indexes of mice in the 3D, oseltamivir, and ribavirin treatment groups were significantly lower than those in the virus control group (Figure 6C), clearly indicating that treatment with drugs could obviously improve lung injury symptoms. The inhibition rate of the 3D high-dose group reached as high as 33.21%, which was similar to the inhibition rates of oseltamivir or ribavirin group (Table 3). Histopathologic examination of lung tissues showed that the lungs of the normal control mice were normal in terms of size, color, and form. However, the lung tissues of the virus control group had severe infiltration of inflammatory cells, thickened alveolar walls, and inflammatory cells that are full of the alveolar cavity. When exposed to 3D, oseltamivir, or ribavirin, the severity of pulmonary inflammation was attenuated (Figure 6D). These results indicate the therapeutic effect of 3D in mice that were infected with A/Weiss/43 H1N1.

### 3.7. Effects of 3D on Influenza A Virus the Replication in Mouse Lungs

**Figure 7:**
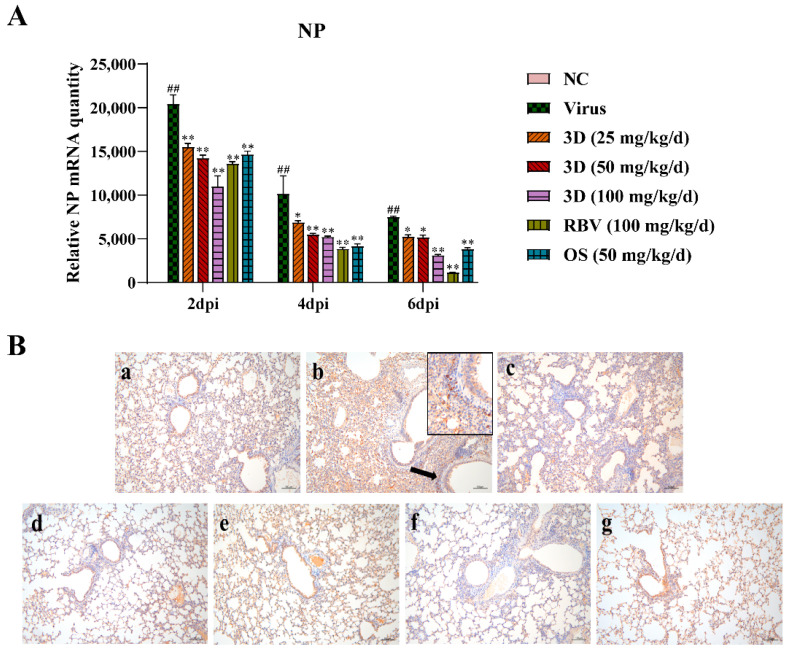
Inhibition of viral replication by 3D in the lung. At two, four and six d days post-infection, mice were euthanized and lung tissues were stored at −80 °C. (**A**) The expression of viral NP mRNA in lungs was determined using qRT-PCR. Data are shown as the mean ± SD. (^##^ *p* < 0.01 compared with the normal control; * *p* < 0.05, ** *p* < 0.01 compared with the virus control) (**B**) The expression of NP protein in lung tissues at six days post-infection was visualized by immunohistochemistry. Samples from (a) the normal control group; (b) the virus control group; (c) the 3D low-dose group, (d) the 3D middle-dose group, (e) the 3D high-dose group, (f) the ribavirin group, and (g) the oseltamivir group.

The level of viral NP mRNA was detected by qRT-PCR assay to assess the effect of 3D on viral replication. Lung tissues were harvested at two, four, and six days post-infection and qRT-PCR assay was performed. The level of viral NP mRNA was the highest at two days post-infection and then gradually decreased at four and six days post-infection (Figure 7A). After the administration of drugs, the viral NP mRNA expression was significantly lower than that in the virus control group. Likewise, 3D decreased the expression of viral NP mRNA in a dose-dependent manner in the IAV infected lungs. An immunohistochemistry assay was performed in the lungs to detect the expression of viral NP protein. Brown-yellow spots indicate positive immune-histochemical staining. NP was found to express strongly in the lungs of mice in the virus control group, and treatment with drugs significantly decreased the area of NP staining (Figure 7B). Taken together, these observations indicate that 3D exerts an inhibitory effect on A/Weiss/43 H1N1 in vivo.

### 3.8. Effects of 3D on Cytokine Production in Mouse Lungs

**Figure 8:**
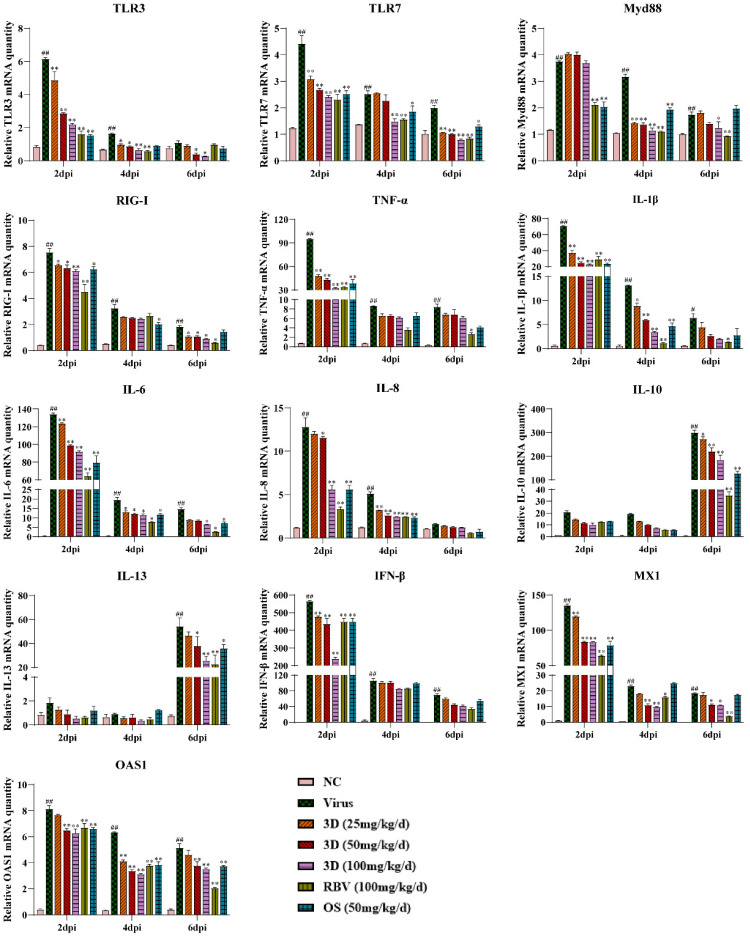
Effects of 3D on the expression of mediators related to RIG-I in vivo. At two, four, and six days post-infection, the mice were euthanized and RNA was isolated from lung tissues. The mRNA expression of the genes expressing indicated mediators in the lungs was determined using qRT-PCR. Data are shown as the mean ± SD. (^#^ *p* < 0.05, ^##^ *p* < 0.01 compared with the normal control; * *p* < 0.05, ** *p* < 0.01 as compared with the virus control).

The effect of 3D on cytokine production in mice lungs was assessed by measuring the levels of several inflammatory cytokines in the lungs at two, four, and six days post-infection. Meanwhile, the mRNA expression levels of genes expressing pattern-recognition receptors (PRRs) that triggered the synthesis and release of cytokines were also assayed. The levels of TLR3, TLR7, myeloid differentiation primary response 88 (Myd88), and RIG-I were up-regulated due to viral infection, which were decreased, as expected, in the presence of 3D, oseltamivir, or ribavirin. We then tested the effect of 3D on the expressions of inflammatory cytokines. The levels of pro-inflammatory cytokines (TNF-α, IL-1β, IL-6, and IL-8) were markedly higher after infection than those in the normal control group. The administration of 3D, oseltamivir, or ribavirin could inhibit the increased expressions of pro-inflammatory cytokines. Unexpectedly, the expression of anti-inflammatory cytokines (IL-10 and IL-13) was also repressed with the treatment of drugs. Interferon-β (IFN-β) is the downstream signaling factor of RIG-I and is widely considered to inhibit viral replication. The level of IFN-β increased significantly after viral infection and reduced after the treatment with 3D, oseltamivir, or ribavirin at two days post infection. On four and six days post infection, there was a decrease in the level of IFN-β in drug-treated groups as compared to that in the virus control group, although the difference is not significant. In addition, the mRNA expression of genes expressing antiviral proteins myxovirus resistance 1 (Mx1) and 29,59-oligoadenylate synthetase 1 (OAS1) was also repressed by the treatment with drugs (Figure 8).

## 4. Discussion

Emerging and re-emerging viruses pose a continuous threat to human health [23]. Influenza viruses, which are a typical example of re-emergence, inflict immense morbidity and mortality that range from 291,000 to 646,000 annual deaths worldwide [24]. The administrations of vaccines and targeted antiviral drugs are restricted due to viral mutations [25,26,27]. With the scarcity of diverse and effective medical methods, a series of new strategies are urgently needed. Based on these reasons, 3D, a novel butene lactone derivative, was synthesized and its antiviral effects were assessed.

Viral load is a key factor in the pathogenesis of influenza [28], so the effect of an anti-viral compound on viral load is assessed in order to measure its efficiency. In this study, 3D exhibited a broad-spectrum antiviral activity against several influenza viruses A/Weiss/43 H1N1, A/Virginia/ATCC2/2009 H1N1 (pdm09), and A/California/2/2014 H3N2 in vitro. The antiviral activity of 3D against A/Weiss/43 H1N1 was confirmed by viral inhibition, viral titers, and cytopathic effect. In addition, 3D significantly repressed the viral genes in MDCK, A549 cells, and mouse lungs. Therefore, 3D could inhibit viral replication in vivo and in vitro and it impelled us to clarify the anti-influenza mechanism of 3D.

The mode of action of 3D in inhibiting IAV replication was examined at three different stages (before, during, and after viral infection). We found that 3D inhibits viral replication in more than one way (during, as well as after viral infection) and significantly prevents the viral infection when administered post-infection. The IAV life cycle is approximately 8–10 h and it includes entry, replication and transcription, virion assembly, and virus budding [29,30]. The time-of-addition assay revealed that 3D inhibited viral replication at the early stage of its life cycle.

Apoptosis, which is a type of programmed cell death, plays a complicated and pivotal role in IAV replication and pathogenicity [31]. Influenza virus induces apoptosis in a variety of cell types. Hosts and viruses regulate apoptosis to achieve their goals. While hosts use apoptosis to resist the invasion of pathogens [32,33], viruses employ apoptosis to evade the host response and accelerate their own replication [34,35]. In the initial stage, IAV suppresses the apoptosis of cells to gain sufficient time for its replication and production of viral proteins [32,36]. In the late stage of infection, IAV induces apoptosis via caspase-3 activation to promote the release of virions and accelerate the infection of cells [37,38]. In our study, 3D was found to suppress the apoptosis in A549 cells induced by A/Weiss/43 H1N1 in a dose-dependent manner at 24 h post-infection and repressed the expression of cleaved caspase-3, caspase-7, caspase-9, and cleaved PARP (the main cleavage target of caspase-3 in vivo). The mitochondrial pathway, as a primary mechanism of apoptosis, is controlled by the Bcl-2 family of protein. Bcl-2 is associated with damaged replication of the influenza virus [39,40]. An increase in the Bax/Bcl-2 ratio indicates the activation of intrinsic apoptosis [41]. We observed that 3D decreased the Bax/Bcl-2 ratio. These results indicate that 3D suppresses apoptosis via the mitochondrial pathway, which may be the mechanism of the anti-IAV effect of 3D.

The first host defense against viral infection is the immune response. Host recognizes the conserved components of pathogens, called pathogen associated molecular patterns (PAMPs). For IAV, double-stranded and triphosphorylated RNAs are PAMPs that are detected by pattern recognition receptors (PRRs), such as TLR3, TLR7, and RIG-I [42,43,44,45]. RIG-I is the primary receptor for recognizing the PAMPs of IAVs in the infected host cells and it has been widely studied [46]. A variety of IFNs and cytokines are released to limit the spread of viruses by interfering with virus release and translation of viral proteins [47]. However, over-production of pro-inflammatory cytokines and chemokines, and immune cell activation may lead to acute lung injury and, thus, has an important role in the pathophysiology of influenza-induced acute respiratory distress syndrome [48]. Pulmonary inflammation and respiratory failure are the primary reasons for the death of patients that are infected with IAV [49]. Therefore, anti-inflammatory treatment for lung injury caused by viruses has shown promise in preclinical models [50]. H5N1 and H1N1 has been found to trigger cells in mice to release inflammatory cytokines and chemokines, including TNF-α, IL-6, IL-1β, IL-8, and IFN-β, which are involved in RIG-I and TLR3 signaling pathways [51,52,53]. These findings suggest that the expression of these pro-inflammatory cytokines is dependent on RIG-I and TLR3. Some anti-viral compounds could reduce the expression levels of inflammatory factors (IL-1, IL-6, and TNF-α, etc.) by suppressing the activation of RIG-I and TLR3 [54,55,56]. Consistent with these findings, our study showed that 3D administration could repress the expression of inflammatory cytokines (IL-6, IL-8, IL-1β, and TNF-α) by inhibiting RIG-I, TLR3, and their downstream signal factors (TLR7, MAVS, Myd88, etc.) in vivo and in vitro. IL-10 and IL-13 have anti-inflammatory properties [57]. The 3D-mediated inhibition of RIG-I and TLR3 expression led to decreased IL-10 and IL-13 levels. Type I interferons (IFN-α and IFN-β) are key innate antiviral defense effectors [58], which are critical for resisting viral invasion. However, recent studies have shown that continuous IFN stimulation can aggravate the expression of cytokines that are mediated by IAV and they contribute to lung injury [44,59]. Congruent with this, our study demonstrates that 3D can down-regulate the expression of IFN-β. Moreover, OAS1 and Mx1 induced by IFNs can restrict viruses [60], and their levels decreased after 3D administration. 3D could also alleviate the severe clinical symptoms (such as inactivity, anorexia, labored breathing, and ruffled fur), moderate the weight loss, reduce the lung index, and mitigate lung damage in infected mice. Therefore, relief from lung damage and protective effect against IAV were associated with the regulation of inflammation by 3D.

## 5. Conclusions

The current study showed the mechanism of action of 3D, a novel compound possessing anti-IAV properties. Our study demonstrates that 3D, which is a butene lactone derivative, exhibits a broad-spectrum antiviral effect against influenza virus, including the 2009 pandemic strain. Additionally, 3D could suppress the apoptosis induced by the influenza virus via the mitochondrial pathway. With the treatment of 3D, the production of inflammatory cytokines was alleviated during viral infection in vivo and in vitro. Further structural optimization and screening of 3D will be carried out in our future work.

## Figures and Tables

**Table 1:** Anti-viral activity against A/Weiss/43 H1N1 of synthetic compounds in Madin–Darby canine kidney (MDCK) cells.

ID	R_1_	R_2_	CC_50_ (µM) ^1^	EC_50_ (µM) ^2^	SI ^3^
3A	*p*-F	H	191.18 ± 3.87	-	-
3B	*p*-F	Ph	677.68 ± 4.05	-	-
3C	*p*-Cl	H	256.25 ± 3.93	-	-
3D	*p*-Cl	Ph	365.02 ± 1.58	12.30 ± 0.53	29.68
3E	*p*-Br	H	225.78 ± 4.09	-	-
3F	*p*-Br	Ph	119.49 ± 1.52	16.15 ± 0.96	7.40
4A	*p*-F	H	256.63 ± 11.69	25.01 ± 0.77	10.26
4B	*p*-F	Ph	614.86 ± 41.20	-	-
4C	*p*-Cl	H	389.22 ± 7.34	22.17 ± 2.65	17.56
4D	*p*-Cl	Ph	338.42 ± 8.14	-	-
4E	*p*-Br	H	238.76 ± 10.70	19.12 ± 2.97	12.49
4F	*p*-Br	Ph	359.72 ± 5.22	-	-

^1^ 50% cytotoxic concentration; ^2^ 50% effective concentration; ^3^ Selectivity index (ratio of CC_50_ to EC_50_).

**Table 2:** Antiviral activities of different drugs against influenza virus (IAV) strains.

Compound	CC_50_ (µM)	A/Weiss/43 H1N1		A/Virginia/ATCC2/2009 H1N1		A/California/2014 H3N2	
		EC_50_ (µM)	SI	EC_50_ (µM)	SI	EC_50_ (µM)	SI
3D	365.02 ± 1.58	12.30 ± 0.53	29.68	18.08 ± 2.60	20.19	24.09 ± 1.16	15.15
Oseltamivir	>800	0.57 ± 0.29	>1403.51	6.97 ± 0.38	>114.78	9.47 ± 2.48	>84.48
Ribavirin	608.33 ± 33.95	22.21 ± 0.76	27.39	13.92 ± 1.06	43.70	12.99 ± 3.74	46.83

**Table 3:** The inhibitory effect of 3D on lung index at sic days post infection.

Group	Mice	Lung Index	Inhibition ^1^
Normal control	10	0.61 ± 0.03	-
Virus control	10	1.70 ± 0.06 ^##^	-
3D (25 mg/kg/d)	10	1.51 ± 0.06	11.35%
3D (50 mg/kg/d)	10	1.22 ± 0.09 **	28.47%
3D (100 mg/kg/d)	10	1.14 ± 0.05 **	33.21%
Ribavirin	10	1.12 ± 0.15 **	34.27%
Oseltamivir	10	1.09 ± 0.12 **	35.99%

^1^ Inhibition ratio of lung index. (^##^ *p* < 0.01 as compared with the normal control; ** *p* < 0.01 compared with the virus control).

## Data Availability

The data presented in this study are available in Supplementary Materials.

## Supplementary Materials

The following are available online at https://www.mdpi.com/1999-4915/13/2/278/s1, Table S1: Primers for qRT-PCR.
