# Supplementary material for: Antiviral Activity of 3D, a Butene Lactone Derivative Against Influenza A Virus In Vitro and In Vivo
Source: Viruses. 2021 Feb 11;13(2):278. doi: 10.3390/v13020278 (PMC7916974; doi:10.3390/v13020278)
Supplement: Supplementary file 1 [file viruses-13-00278-s001.pdf]

viruses

MDPI

Supplementary Materials: Antiviral Activity of 3D, a Butene Lactone Derivative Against Influenza A Virus In Vitro and In Vivo

Table S1. Primers for qRT-PCR.

Gene	Primer pairs (5'–3')
IAV-M	F: AGATGAGTCTTCTAACCGAGGTCG<br>R: TGCAAAAACATCTTCAAGTCTCTG
IAV-NP	F: CAAGGCACCAAACGGTCTTAC<br>R: TTGGATGTAGAATCGCCCAAT
GAPDH (D)[1]	F: AGTCAAGGCTGAGAACGGGAAACT<br>R: TCCACAACATACTCAGCACCAGCA
TNF-α (H)[2]	F: AGCAACAAGACCACCACTTCG<br>R: TCAGGGATCAAAGCTGTAGGC
IL-6 (H)	F: TGCAATAACCACCCCTGACC<br>R: TGCGCAGAATGAGATGAGTTG
IL-8 (H)	F: TGCAGAGGGTTGTGGAGAAGT<br>R: AAGGCAGATACCTAATGACGA
IL-1β (H)	F: AGCTACGAATCTCCGACCAC<br>R: CGTTATCCCATGTGTCGAAGAA
RIG-I (H)	F: CCAAGCCAAAGCAGTTTTCAAG<br>R: CATGGATTCCCCAGTCATGG
TLR3 (H)	F: CTTCAACGACTGATGCTCCG<br>R: GCTTGACAGCTCAGGGATG
IRF3 (H)	F: ACCAGCCGTGGACCAAGAG<br>R: TACCAAGGCCCTGAGGCAC
IRF7 (H)	F: TCCCCACGCTATACCATCTACCT<br>R: ACAGCCAGGGTTCCAGCTT
MAVS (H)	F: TGATTTCCTCGCAATCAGACG<br>R: GAAGCCGATTTCCAGCTGTATG
GAPDH (H)	F: GCACCGTCAAGGCTGAGAAC<br>R: TGGTGAAGACGCCAGTGGA
TLR3 (M)[3]	F: AAAGGGTGTTCCTCTTATC<br>R: AAGTTGGTAGGTGGTAATC
TLR7 (M)	F: CTTGACCTAAGTGGAAATTG<br>R: CATGCTGAAGAGAATTACTG
MyD88 (M)	F: AAGGCGATGAAGAAGGAC<br>R: CATTGAACACGGGTTGAG
RIG-I (M)	F: ATCTGTAAACTCTGTGCCGCC<br>R: CCTCGGAGACATCCTTGGCT
TNF-α (M)	F: CGGTGCCTATGTCTCAGCCT<br>R: GCCTTGTCCCTTGAAGAGAACC
IL-1β (M)	F: AAAGCCTCGTGCTGTCGGA<br>R: CTCTGCTTGTGAGGTGCTGATGTA
IL-6 (M)	F: GTTGCCTTCTTGGGACTGATG<br>R: CTCATTTCCACGATTTCCCAGA
IL-8 (M)	F: CCTTGTGGGGAACTCCTTGG<br>R: GCAGGTAGACATCGGTGACAGA
IL-10 (M)	F: ATCACACAAGACCAGACTCCC<br>R: CTCTGGGTCCTGTAGATGGC
IL-13 (M)	F: ATCACACAAGACCAGACTCCC<br>R: CTCTGGGTCCTGTAGATGGC
IFN-β (M)	F: CTGCTGTGCTTCTCCACCAC

	R: TTGAAGTCCGCCCTGTAGGT
MX1 (M)	F: TGGTAGTCCCCAGCAATGTG<br>R: TTCCGCATCACATCCAAGAC
OAS1 (M)	F: GTCTTTGCCTGGGAACAAGG<br>R: GCCTGGCTTTTCTGAGCTGT
GAPDH (M)	F: ATGACATCAAGAAGGTGGTG<br>R: CATACCAGGAAATGAGCTTG

[1] Primers specific for dog genome; [2] Primers specific for *Homo sapiens*; [3] Primers specific for mice.
